# Clinical symptoms of androgen deficiency in men with migraine or cluster headache: a cross-sectional cohort study

**DOI:** 10.1186/s10194-021-01334-3

**Published:** 2021-10-19

**Authors:** Iris E. Verhagen, Roemer B. Brandt, Carlijn M. A. Kruitbosch, Antoinette MaassenVanDenBrink, Rolf Fronczek, Gisela M. Terwindt

**Affiliations:** 1 Department of Neurology, Leiden University Medical Center, P.O. 9600, 2300 WB Leiden, the Netherlands; 2 Department of Internal Medicine, Erasmus University Medical Center, Rotterdam, The Netherlands

**Keywords:** Headache, Morning erections, Libido, Sexual potency, Sex hormones

## Abstract

**Background:**

To compare symptoms of clinical androgen deficiency between men with migraine, men with cluster headache and non-headache male controls.

**Methods:**

We performed a cross-sectional study using two validated questionnaires to assess symptoms of androgen deficiency in males with migraine, cluster headache, and non-headache controls. Primary outcome was the mean difference in androgen deficiency scores. Generalized linear models were used adjusting for age, BMI, smoking and lifetime depression. As secondary outcome we assessed the percentage of patients reporting to score below average on four sexual symptoms (beard growth, morning erections, libido and sexual potency) as these items were previously shown to more specifically differentiate androgen deficiency symptoms from (comorbid) anxiety and depression.

**Results:**

The questionnaires were completed by *n* = 534/853 (63%) men with migraine, *n* = 437/694 (63%) men with cluster headache and *n* = 152/209 (73%) controls. Responders were older compared to non-responders and more likely to suffer from lifetime depression.

Patients reported more severe symptoms of clinical androgen deficiency compared with controls, with higher AMS scores (Aging Males Symptoms; mean difference ± SE: migraine 5.44 ± 0.90, *p* <  0.001; cluster headache 5.62 ± 0.99, *p <*  0.001) and lower qADAM scores (quantitative Androgen Deficiency in the Aging Male; migraine: − 3.16 ± 0.50, *p <*  0.001; cluster headache: − 5.25 ± 0.56, *p <*  0.001). Additionally, both patient groups more often reported to suffer from any of the specific sexual symptoms compared to controls (18.4% migraine, 20.6% cluster headache, 7.2% controls, *p* = 0.001).

**Conclusion:**

Men with migraine and cluster headache more often suffer from symptoms consistent with clinical androgen deficiency than males without a primary headache disorder.

## Introduction

Migraine and cluster headache are primary headache disorders that share certain pathophysiological characteristics, but have a very different epidemiology and phenotype. Migraine prevalence is three times higher in women than in men [1]. Fluctuations in female sex hormones during the menstrual cycle and menopausal transition are associated with an increase in attack susceptibility, while pregnancy and postmenopausal status are associated with a decrease [2–4]. With migraine being a predominantly female disease, a limited number of studies has investigated sex hormones in men, but one small scale study showed a decreased testosterone/estradiol ratio in males with migraine [5].

As opposed to migraine, cluster headache was historically considered a male disease. Recent studies show that cluster headache occurs in women more often than previously assumed with a male to female ratio of 2:1 [6]. Onset of cluster headache before puberty is rare and cluster headache patients have been characterized as over-masculinized [7]. These clinical observations may suggest a role for androgens in cluster headache pathophysiology, but several small studies evaluating androgens, and testosterone in particular, have led to conflicting results.

Calcitonin gene-related peptide (CGRP) is known to be involved in the pathophysiology of migraine and cluster headache. Studies have indicated that (fluctuations in) sex hormones can modulate CGRP in the trigeminovascular system [8]. Sex hormones might thus play a role through CGRP. As relative androgen deficiency has been suggested in men with migraine, but may play a role in cluster headache as well, we aimed to compare symptoms of clinical androgen deficiency between male migraine and cluster headache patients and controls.

## Methods

### Study design and participants

We performed a cross-sectional questionnaire study among men with migraine, men with cluster headache and male controls without headache. Data were collected between October 2019 and April 2020. Participants were selected from the Leiden University Migraine Neuro Analysis (LUMINA) and the Leiden University Cluster headache neuro Analysis (LUCA) cohort [9, 10]. Men with migraine (episodic or chronic) or cluster headache (episodic or chronic) that fulfilled the International Classification of Headache Disorders (ICHD-3) criteria, and men without a primary or secondary headache disorder (apart from an occasional episodic tension-type headache), who gave written informed consent to be contacted in case of future research, were identified.

This study was approved by the medical ethics committee of Leiden University Medical Center. All subjects provided written informed consent.

### LUMINA and LUCA program

Migraine patients, cluster headache patients and controls aged 18-80 years were recruited via nationwide public announcement, advertising in lay press and our research website. They were considered eligible after a two-step inclusion process using validated questionnaires. Additionally, patients attending our outpatient headache clinic were invited to participate. Migraine and cluster headache patients were first asked to fill out a validated web-based screening questionnaire with a sensitivity of 0.93 and specificity of 0.36 for migraine, and a sensitivity of 1.00 and specificity of 0.58 for cluster headache [1, 10]. Patients who fulfilled the screening criteria for migraine or cluster headache, were sent a validated web-based extended migraine or cluster headache questionnaire, based on the International Classification of Headache Disorders criteria (previously ICHD-2, now ICHD-3 version) criteria [11]. The specificity of the extended migraine questionnaire was 0.95 and sensitivity was 0.45 [9]. This questionnaire is described in detail elsewhere [9]. The extended cluster headache questionnaire has a diagnostic specificity of 0.88 and sensitivity of 0.57 [10, 11].

We consider the cohort a well-defined web-based cohort. Four percent of migraine patients were included from our headache outpatient clinic and 87% of the participants were previously diagnosed with migraine by a physician. A clinically confirmed diagnosis of cluster headache by a physician was available for 94% of the LUCA population [10]. The LUCA questionnaire was primarily validated for the ICHD-II criteria for cluster headache. However, all patients who fulfilled the ICHD-II criteria fulfilled the ICHD-III criteria for cluster headache as well [12]. In addition to questions that were necessary to diagnose migraine and cluster headache accurately, the extended questionnaires also included items on demographic factors, aura and headache characteristics, acute and prophylactic headache medication use, and allodynia. Participants unable to use the web-based questionnaires due to lack of the needed internet skills were allowed to fill out the questionnaires on paper.

### Questionnaires

Eligible subjects were invited to complete an online questionnaire with general questions about BMI, smoking and the use of (anti) androgen medication, and two validated questionnaires to assess clinical androgen status. In addition, patients provided migraine- or cluster headache specific information, whereas non-headache controls verified that they had not developed migraine or cluster headache since signing up as a control. Subjects using (anti) androgen medication and controls who had developed migraine or cluster headache were later excluded from all analyses. Reminders were sent out thrice to all subjects who had not yet participated.

Two questionnaires were used to assess clinical androgen status: the Aging Males’ Symptoms (AMS) scale, which consists of 17 items rated from 1 (none) to 5 (extremely severe) [13] and the quantitative Androgen Deficiency of Ageing Men (qADAM) questionnaire including 10 items scored on a Likert scale from 1 (absence of symptom) to 5 (maximal symptoms). The AMS scale is used to assess symptoms of aging across multiple countries and has an overall test-retest stability of 0.86 [14]. The qADAM questionnaire is adapted from the ADAM questionnaire which includes the same 10 items only dichotomized [15]. Both AMS and qADAM total scores are shown to be correlated with serum testosterone levels [15, 16]. A higher AMS score corresponds with lower serum testosterone levels, while lower qADAM scores correspond with lower serum testosterone levels.

### Statistical analyses

Primarily, generalized linear models were fitted to assess the mean difference in AMS and qADAM scores between men with migraine, cluster headache and non-headache controls. Since age, BMI and smoking are associated with alterations in the hypothalamic-pituitary-testicular axis and a consequent decline in testosterone, age, BMI, smoking and lifetime depression were included as covariates [17]. Age and BMI were treated as continuous variables, smoking as dichotomous variable and lifetime depression as categorical variable. Lifetime depression was defined as a HADS-D ≥ 8 or CES-D ≥ 16 or (past/present) depression diagnosed by a physician or (past/present) use of antidepressants for depression [18]. For some participants data on lifetime depression were missing, for whom it was set to unknown and analyzed as such.

As both the AMS and qADAM questionnaire contain multiple items associated with anxiety and depression, which are more prevalent in chronic headache disorders, we performed a secondary analysis determining the number of patients reporting to score below average (≥4) on four selected items of the AMS scale regarding sexual symptoms (beard growth, morning erections, libido and sexual potency). In an earlier study these items were shown to differentiate aging symptoms from anxiety and depression [19]. Percentages of patients scoring below average were calculated for each group using descriptive statistics.

Additionally, exploratory analyses were performed for episodic versus chronic migraine and episodic versus chronic cluster headache. Chronic migraine was defined as an average of ≥15 headache days per month during the past 3 months, from which ≥8 days fulfil criteria for a migraine attack according to ICHD-3 [20]. Chronic cluster headache was defined as remission periods lasting < 3 months for at least 1 year according to ICHD-3 [20]. Furthermore, to rule out the influence of prophylactic medication, subgroup analyses were performed in migraine patients who did not use any prophylactic medication, and in episodic cluster headache patients out of bout.

Two-sided *p*-values < 0.05 were considered statistically significant. Due to a clear distinction between primary and exploratory analyses, no corrections for multiple testing were made [21]. All analyses were performed in R version 3.6.1

## Results

We sent an invitation to participate in this specific study to *n* = 853 migraine patients, *n* = 694 cluster headache patients, and *n* = 209 controls without headache. Questionnaires were completed by 534/853 (63%) migraine patients, 437/694 (63%) cluster headache patients and 152/209 (73%) controls. Responders were slightly older than non-responders and suffered less often from lifetime depression. No other differences were found between responders and non-responders for all three groups.

Patients with migraine, cluster headache and non-headache controls were comparable regarding age and BMI. Patients with migraine and cluster headache were more likely to suffer from lifetime depression than healthy controls as was expected (36.7% and 36.6% vs. 9.9%). Patients with cluster headache smoked more often than healthy controls (57.7% vs. 9.2%), while patients with migraine smoked less often than controls (5.4% vs. 9.2%). Baseline characteristics are shown in Table 1.

**Table 1:** Baseline characteristics for each group

	Migraine	Cluster headache	Controls
Number of patients, n	534	437	152
Age (years), mean ± SD	52.43 ± 13.47	53.23 ± 12.92	54.16 ± 13.76
BMI (kg/m^2^), mean ± SD	25.43 ± 3.54	25.43 ± 3.62	25.12 ± 3.12
Smoking, n (%)	29 (5.4)	251 (57.7)	14 (9.2)
Lifetime depression, n (%)			
Yes	196 (36.7)	160 (36.6)	15 (9.9)
No	274 (51.3)	198 (45.3)	132 (86.8)
Unknown	64 (12.0)	79 (18.1)	5 (3.3)
Chronic migraine, n (%)	82 (15.4)	NA	NA
Chronic CH, n (%)	NA	99 (22.7)	NA

*Abbreviations*: *BMI* body mass index, *SD* standard deviation, *CH* Cluster headache

Chronic migraine was defined based on the ICHD-3 criteria as ≥15 headache days per month, from which ≥8 migraine days. Chronic cluster headache was defined based on the ICHD-3 criteria as remission periods lasting < 3 months for at least 1 year. Lifetime depression was defined as a HADS-D ≥ 8 or CES-D ≥ 16 or (past) depression diagnosed by a physician or (past) use of antidepressants for depression

**Fig. 1:**
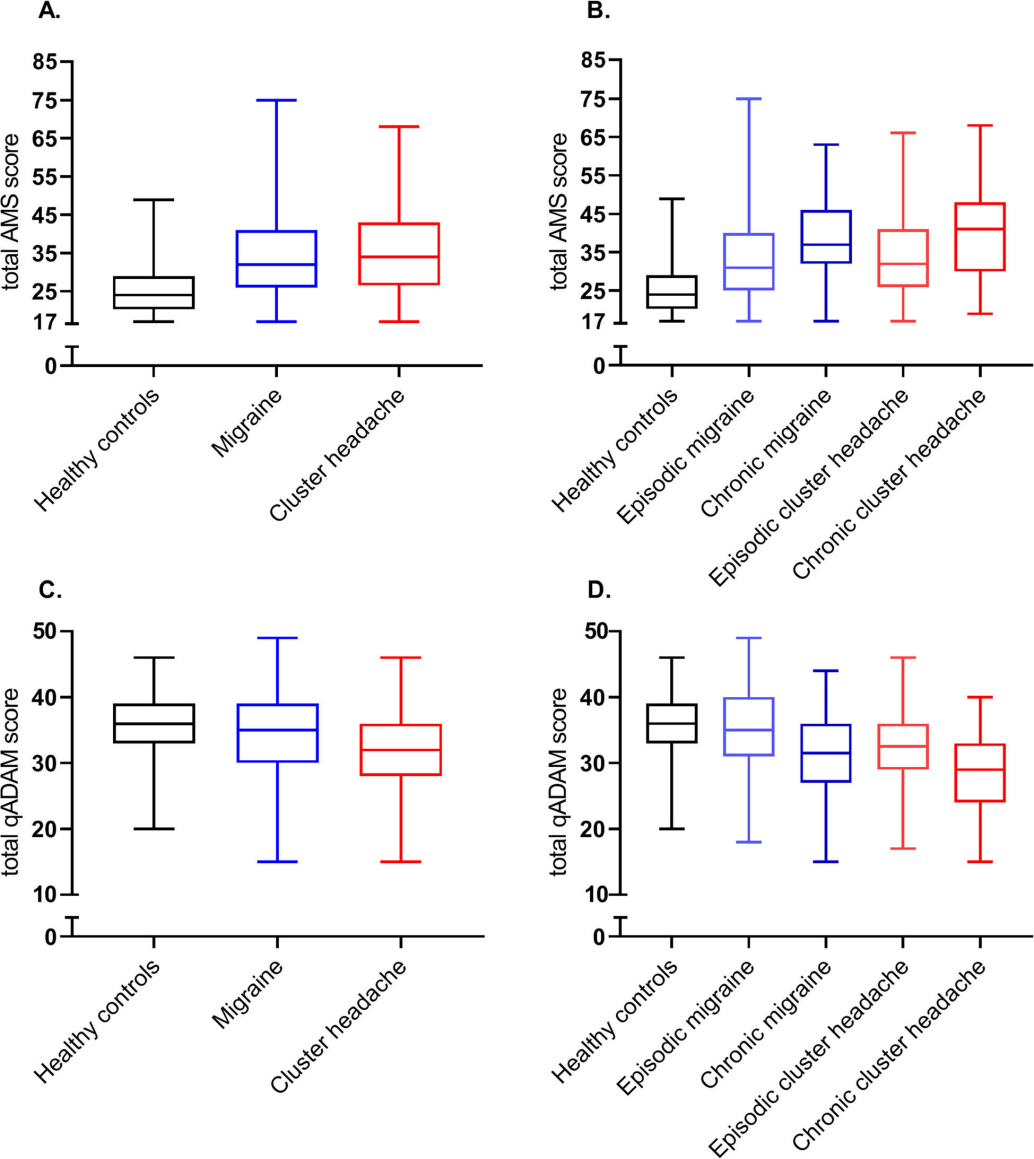
Crude median total scores on two validated clinical androgen deficiency questionnaires (AMS and qADAM) for male migraine patients, male cluster headache patients and male non-headache controls, Boxplot with whiskers for minimum and maximum score. Abbreviations: AMS = Aging Males Symptoms. qADAM = quantitative Androgen Deficiency in the Aging Male. Episodic and chronic migraine and cluster headache were defined based on the ICHD-3 criteria as ≥15 headache days per month, from which ≥8 migraine days, and remission periods lasting < 3 months for at least 1 year, respectively [20]

In the crude data, both migraine and cluster headache patients scored higher on the AMS (the higher the score, the more androgen deficiency symptoms) and lower on the qADAM scale (a lower score indicates more symptoms) than healthy controls, corresponding with more severe symptoms of clinical androgen deficiency. Patients with chronic migraine or cluster headache scored higher on the AMS and lower on the qADAM than episodic patients. Box plots showing the crude data can be found in Fig. 1.

### Primary analysis

After adjusting for age, BMI, smoking and lifetime depression, differences were found in both AMS and qADAM scores between men with migraine, cluster headache and non-headache controls (see Tables 2 and 3). The mean AMS scores were higher in patients with migraine compared to controls (mean difference ± SE: 5.44 ± 0.90, *p <*  0.001), and cluster headache compared to controls (5.62 ± 0.99, *p <*  0.001). Mean qADAM scores were lower in patients with migraine compared to controls (− 3.16 ± 0.50, *p <*  0.001), and cluster headache compared to controls (− 5.25 ± 0.56, *p <*  0.001).

**Table 2:** Results of generalized linear model with total AMS scale as dependent variable and headache type (control versus migraine versus cluster headache) as independent variable, corrected for age, BMI, smoking and lifetime depression. Control was set as the reference group

	β	SE	*p-*value
Intercept	13.73	2.37	< 0.001
Headache type			
Migraine	5.44	0.90	< 0.001
Cluster headache	5.62	1.00	< 0.001
Age	0.02	0.02	0.28
BMI	0.37	0.08	< 0.001
Smoking	2.05	0.79	0.01
Lifetime depression			
Yes	9.67	0.65	< 0.001
Unknown	4.31	0.90	< 0.001

**Table 3:** Results of generalized linear model with total qADAM score as dependent variable and headache type (control versus migraine versus cluster headache) as independent variable, corrected for age, BMI, smoking and lifetime depression. Control was set as the reference group

	β	SE	*p*-value
Intercept	47.09	1.32	< 0.001
Headache type			
Migraine	−3.16	0.50	< 0.001
Cluster headache	−5.25	0.56	< 0.001
Age	−0.06	0.01	< 0.001
BMI	−0.18	0.05	< 0.001
Smoking	−1.88	0.44	< 0.001
Lifetime depression			
Yes	−4.53	0.36	< 0.001
Unknown	−1.26	0.50	0.012

### Exploratory analyses

Subgroup analysis in migraine patients not using prophylactic medication (*n* = 242) compared to controls revealed similar results after correction for potential confounders for AMS total score (mean difference ± SE: 5.37 ± 0.93, *p* <  0.001), and qADAM total score (mean difference ± SE: − 2.59 ± 0.57, *p <*  0.001). The number of monthly migraine days was weakly correlated with both AMS total score (*r =* 0.21, 95% CI: 0.13 to 0.29, *p <*  0.001), and qADAM total score (*r =* − 0.27, 95% CI: − 0.34 to − 0.19, *p* <  0.001).

After correction for potential confounders, AMS total scores were higher in men with episodic cluster headache during a bout (*n* = 47) than out of bout (*n* = 291) (mean difference ± SE: 4.76 ± 1.58, *p* = 0.007), while qADAM total scores were lower (− 3.13 ± 0.84, *p <*  0.001). Men with episodic cluster headache out of bout still scored higher on the AMS (4.72 ± 1.04, *p <*  0.001) and lower on qADAM (− 4.62 ± 0.58, *p <*  0.001) than non-headache controls. No significant differences were found between men with episodic cluster headache during a bout and men with chronic cluster headache (AMS: − 0.22 ± 1.65, *p* = 0.89 / qADAM: 0.22 ± 0.91, *p* = 0.81).

**Fig. 2:**
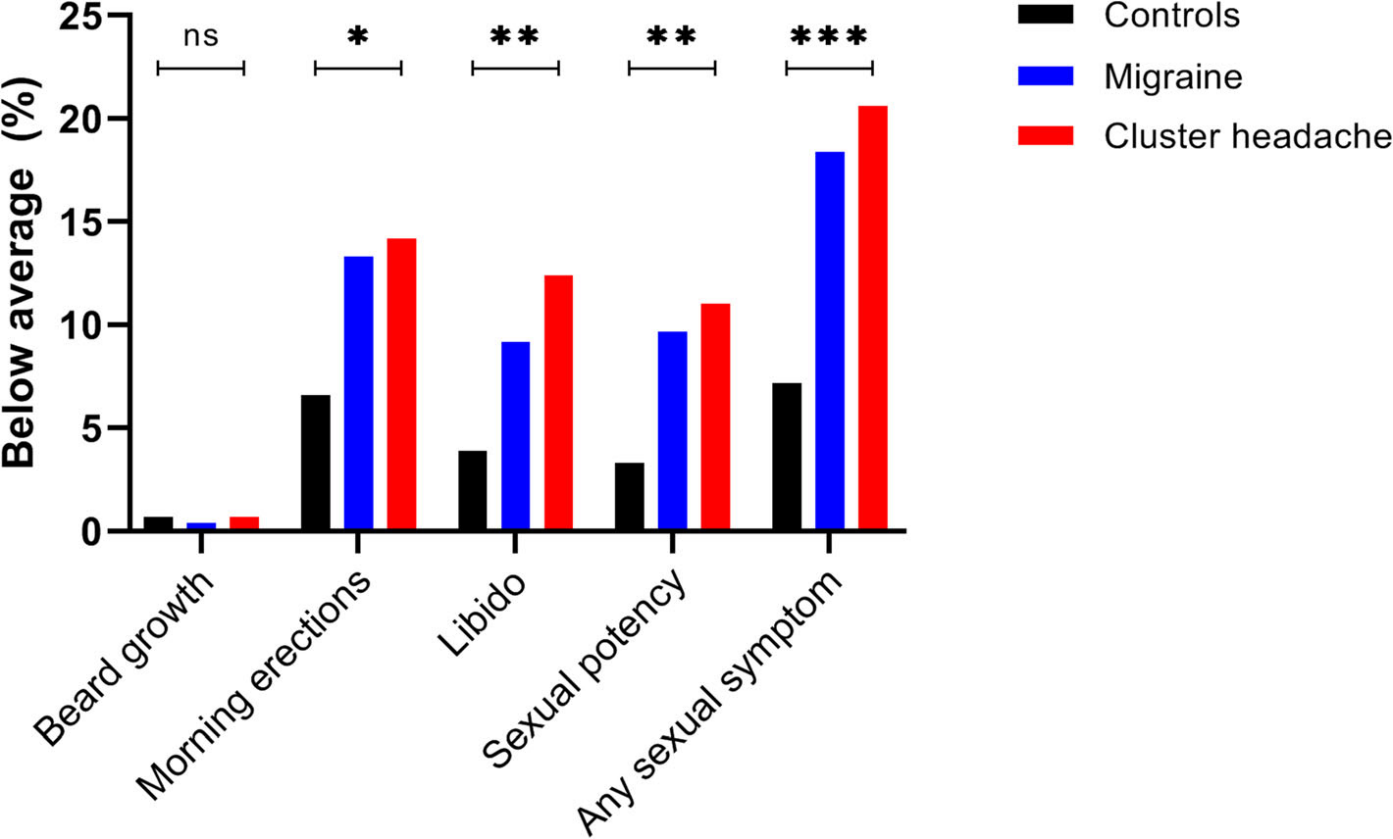
The percentage of male migraine patients, male cluster headache patients and male non-headache controls reporting to score below average on each of the individual items assessing sexual symptoms, and the percentage of participants suffering from diminishment of at least one of these four sexual symptoms. Abbreviations: NS = not significant; * = *p-*value < 0.05; ** = *p*-value < 0.01; *** = *p-*value < 0.001

**Fig. 3:**
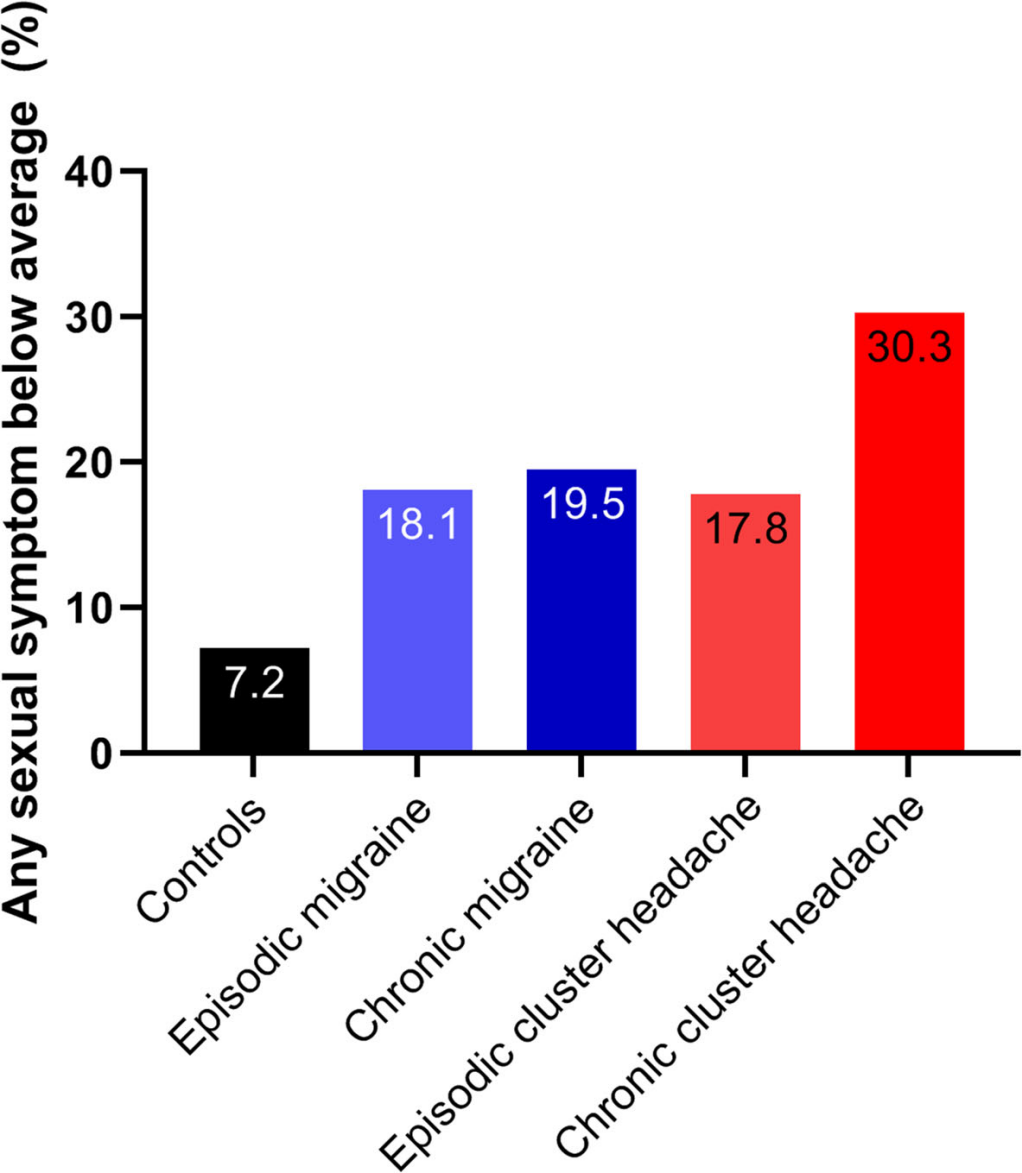
The percentage of male migraine patients, male cluster headache patients and male non-headache controls reporting to score below average on at least one of four specific sexual symptoms; beard growth, morning erections, libido and sexual potency. Abbreviations: AMS = Aging Males Symptoms. qADAM = quantitative Androgen Deficiency in the Aging Male. Episodic and chronic migraine and cluster headache were defined based on the ICHD-3 criteria as ≥15 headache days per month, from which ≥8 migraine days, and remission periods lasting < 3 months for at least 1 year, respectively [20]

Additionally, migraine and cluster headache patients more often reported to score below average on each of the individual sexual items: decreased morning erections, libido and sexual potency, but not beard growth (see Fig. 2). Furthermore, more than twice as many patients with migraine (18.4%) and cluster headache (20.6%) reported to suffer from diminishment of at least one of these four sexual symptoms, compared with non-headache controls (7.2%). Post-hoc comparisons showed that statistically significant differences were present between men with migraine and non-headache controls (except for decreased libido), as well as between patients with cluster headache and non-headache controls (data not shown). Patients with chronic migraine (15% of migraine participants) and chronic cluster headache (23% of cluster headache participants) more often reported a diminishment of at least one sexual symptom (see Fig. 3 and Table 4).

**Table 4:** The percentage of male migraine patients, male cluster headache patients and male non-headache controls reporting to score below average on each of the individual items assessing sexual symptoms and the percentage of participants suffering from diminishment of at least one of these four sexual symptoms

	Controls	Migraine	Cluster headache	*p*-value
n	152	534	437	
Beard growth	1 (0.7)	2 (0.4)	3 (0.7)	0.782
Morning erections	10 (6.6)	71 (13.3)	62 (14.2)	0.046
Libido	6 (3.9)	49 (9.2)	54 (12.4)	0.009
Sexual potency	5 (3.3)	52 (9.7)	52 (11.9)	0.008
Any sexual symptom	11 (7.2)	98 (18.4)	90 (20.6)	< 0.001

## Discussion

This cross-sectional study shows that men with migraine and cluster headache more often suffer from symptoms consistent with (relative) clinical androgen deficiency than males without a primary headache disorder. In addition, our study shows that these symptoms are more pronounced in men with chronic migraine, i.e. a high attack frequency, and chronic cluster headache, i.e. short or no remission periods.

Androgen deficiency is associated with a wide range of symptoms, ranging from mood disturbances to sexual symptoms such as decreased libido and number of morning erections. Our study shows that patients with migraine and cluster headache suffer more frequently and more severely from these symptoms than men without headache. Whether this is the result of hormonal imbalances or an epiphenomenon reflecting chronic disease remains to be elucidated.

Only limited evidence exists on hormone levels in men with migraine and cluster headache. A recent study in male migraine patients showed symptoms of a relative androgen deficiency with a higher estradiol/testosterone ratio compared to controls, which was attributed to higher estradiol levels [5]. No significant differences were found in free testosterone levels. In contrast, one very small-scale study described low testosterone levels in men with chronic migraine compared to age-matched normative values [22]. Similarly, in contrast to the concept of ‘over-masculinization’, several older studies suggest a lower testosterone level in men with cluster headache [23–27]. These presumed differences in estradiol/testosterone ratios may be responsible for the symptomatology described in the present study, which would strengthen the hypothesis of hormonal imbalances in men with migraine and cluster headache.

As scientific literature on hormone levels in men with primary headache disorders is scarce, it remains unknown whether hormone imbalances contribute to migraine or cluster headache pathophysiology, or that these observations are the result of an (even possibly unrelated) epiphenomenon. An interesting study in male-to-female transsexuals who use antiandrogens and estrogens reported migraine rates similar to genetic females, adding to the notion that sex hormones may contribute to migraine prevalence and possibly migraine pathophysiology [28]. Another disease-modifying effect of androgens was reported in two small scale trials in women in whom improvement in migraine was reported after either implantation of a testosterone pellet or the use of danazol, a steroid hormone [29, 30]. Positive effects of testosterone replacement therapy in a very small group of cluster headache patients was reported as well [31**]**.

A major limitation of the current study is that we used questionnaires as an indirect measure of hormone levels. Therefore, it may be argued that the symptomatology described may not be very specific, and might also be attributed to long-term effects of a chronic disease status, instead of (relative) androgen deficiency. Previous studies have shown that both AMS and qADAM questionnaires display a (moderate) correlation with serum testosterone levels [14, 15]. However, these questionnaires were developed and validated in the aging male and have not been validated in other patient groups such as headache patients. Both questionnaires contain multiple items that are known to be associated with chronic headache disorders such as mood disturbances, anxiety and sleeping problems. We corrected for these confounding factors by including them as covariates in our analyses [32–37]. After correction, differences between headache patients and controls remained present. In addition we performed a secondary analysis, incorporating more robust items that were previously shown to differentiate androgen deficiency symptoms from anxiety and depression [19, 38–42]. This secondary analysis showed that patients with migraine and cluster headache more often report impaired sexual symptomatology, which more specifically points in the direction of androgen deficiency.

Furthermore, the symptomatology described in this study could partly be explained by side effects of prophylactic medication. However, a subgroup analysis in migraine patients who did not use any prophylactic drugs and episodic cluster headache patients out of bout, revealed similar results, making a (large) confounding effect unlikely. We therefore assume that the use of prophylactic drugs has a negligible influence on our final results.

In conclusion, our study shows that men with migraine and cluster headache more frequently report symptomatology consistent with androgen deficiency than males without a primary headache disorder. We hypothesize that these differences may be the result of a (relative) androgen deficiency, which can be attributed to either higher estradiol or lower testosterone levels. However, suffering from a chronic (headache) disorder might be implicated as well. Regardless of the cause, it is important to recognize this symptomatology in patients since this may negatively impact quality of life. We advise physicians to proactively assess mood and sexual symptoms in patients with migraine and cluster headache.

## Data Availability

The datasets used and/or analysed during the current study are available from the corresponding author on reasonable request.

## Abbreviations

AMS: Aging Males Symptoms
qADAM: Quantitative Androgen Deficiency in the Aging Male
