# Correction to: Clinical symptoms of androgen deficiency in men with migraine or cluster headache: a cross-sectional cohort study

**DOI:** 10.1186/s10194-021-01346-z

**Published:** 2021-11-09

**Authors:** Iris E. Verhagen, Roemer B. Brandt, Carlijn M. A. Kruitbosch, Antoinette MaassenVanDenBrink, Rolf Fronczek, Gisela M. Terwindt

**Affiliations:** 1 Department of Neurology, Leiden University Medical Center, P.O. 9600, 2300 Leiden, WB The Netherlands; 2 Department of Internal Medicine, Erasmus University Medical Center, Rotterdam, The Netherlands

**Correction to: J Headache Pain 22, 125 (2021)**

**http://orcid.org/10.1186/s10194-021-01334-3**

Following the publication of the original article [1], we were notified of a few corrections:

1. The corresponding author should be Gisela M. Terwindt, instead of Iris E. Verhagen.
2. In the Results section of the Abstract, the phrase “Responders were older compared to non-responders and more likely to suffer from lifetime depression” should read “Responders were older compared to non-responders and less likely to suffer from lifetime depression”.
3. ICHD-II and ICHD-III were modified into ICHD-2 and ICHD-3.

The original article has been corrected.
